# A novel process to improve the characteristics of low‐fat ice cream using date fiber powder

**DOI:** 10.1002/fsn3.2159

**Published:** 2021-05-01

**Authors:** Ali I. A. Mansour, Mahmoud A. Ahmed, Mohamed Salem Elfaruk, Khalid A. Alsaleem, Ahmed R. A. Hammam, Yaser M. A. El‐Derwy

**Affiliations:** ^1^ Dairy Science Department Faculty of Agriculture Al‐Azhar University Assiut Egypt; ^2^ Dairy and Food Science Department South Dakota State University Brookings SD USA; ^3^ Medical Technology College Nalut University Nalut Libya; ^4^ Department of Food Science and Human Nutrition College of Agriculture and Veterinary Medicine Qassim University Buraidah Saudi Arabia; ^5^ Dairy Science Department Faculty of Agriculture Assiut University Assiut Egypt

**Keywords:** dates fiber powder, low‐fat ice cream, rheological characteristics, sensory quality, shelf‐life

## Abstract

The objective of this study was to improve the characteristics of low‐fat ice cream (LFIC) using date fiber powder (DFP). DFP was added to LFIC mix (3% fat, 14% milk solids nonfat, 15% sucrose, 0.3% stabilizer, and 0.1% vanilla) at a rate of 1.5%, 2.5%, and 3.5%. Control treatment with no DFP was also manufactured for comparison. The LFIC mix was analyzed for physicochemical and microbiological analyses. After manufacture, microbiological, rheological, and sensory characteristics of LFIC were evaluated during storage at −18˚C for 30 days. The addition of DFP to the LFIC mix led to increasing (*p *< .05) the density and weight per gallon (lb) of final product. Thus, a 3.5% of DFP led to increasing the density of LFIC from 0.6 to 1.0 g/cm^3^ and weight per gallon from 5.2 to 9.0 lb, while the overrun of LFIC was decreased (*p *< .05) from 50.0% to 24.0%. Additionally, the melting resistance of LFIC made with DFP was higher (*p *< .05) as compared to control. Approximately 60% of LFIC made with DFP was melted after 50 min compared to 100% in control. The total bacterial count (TBC) and yeast and molds' count slightly increased in LFIC with adding DFP. However, there was a slight decrease in these counts during storage for 30 days. Psychrotrophic and coliform bacteria were not detected in the LFIC. Organoleptically, LFIC made with DFP showed higher scores (*p *< .05) of body and texture, melting quality, and appearance as compared to control during the 30 days of storage. However, the flavor was slightly decreased (*p *< .05) as the concentration of DFP was increased. The overall scores were increased with increasing the DFP concentrations up to 15 days as compared to control, followed by a decrease at 30 days of storage.

## Graphical abstract

This work concluded that date fibers could be used to improve the functionality and properties of low‐fat ice cream to match the properties of full‐fat ice cream with more health benefits.

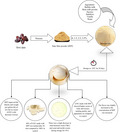

## 1 INTRODUCTION

The standard constituents used to make ice cream are milk solids (fat not included), fat (dairy or nondairy), water, sweeteners, flavors, emulsifiers, and stabilizers. Consequently, there are many types of ice cream including nonfat, low, regular, or high fat, and no‐sugar‐added (sugar‐free forms) (Goff & Hartel, 2013). The quality of ingredients is vital to make high‐quality ice cream. Thus, the quality and type of milk employed in making ice cream have the main role in the final product's properties. In addition, the used production techniques could impact the physical characteristics of the ice cream. Consumers evaluate ice cream quality based on different factors, such as flavors and texture. For instance, the content of total solids in ice cream has a crucial impact on the allocation and volume of ice crystals in which low solids content results in big ice crystals. The global demand for ice cream led ice cream manufacturers to provide customers with various flavors of ice cream. To differentiate ice cream products and compete for market share, some fruits (pineapple, strawberries, and mango), fruit juice (kiwi juice and grape juice), and fruit fibers (date fiber and citrus fiber) were added to ice cream (Gheisari et al., 2020; Hashim & Shamsi, 2016).

Dates are valuable sources of salts and minerals, vitamins, and carbohydrates of both oligosaccharides and polysaccharides (cellulose, hemicelluloses, pectin substances, gums, resistant starch, inulin) (Elleuch et al., 2011), dietary fibers, fatty acids, antioxidants, amino acids, and protein (Kchaou et al., 2013). Therefore, they have high nutritional value and potential health benefits, including enhancing the metabolism of the human body (Elleuch et al., 2011), laxation, blood cholesterol attenuation, and blood glucose attenuation. Different varieties of dates vary in their chemical composition, especially in sugars and dietary fibers content (Singh et al., 2013). Many studies reported that insoluble fibers represent the majority of fiber in dates. Normally, dates comprise about 8 g/100 g total dietary fibers (Nasir et al., 2015) in which 5.76 g/100 g insoluble fiber (Nasir et al., 2015). Since 25 g/day is the recommended daily intake (RDI) of dietary fiber, dates are deemed a good source of them. Insoluble dietary fibers have a vital physiological function in the human's body because they can help to improve defecation (boost the amount of excrement and make it softer), so they assist with preventing some diseases as intestines cancer and diverticular disease (Nasir et al., 2015).

Recently, people are increasingly changing their diet patterns and seeking diets with potential health benefits. Therefore, high‐fiber diets are in great demand in the marketplace due to their health impacts. Not much research has been done on the functionality of date fibers as an ingredient in low‐fat ice cream. Thereupon, this research studied the effects of adding different ratios of data fiber powder (DFP) on low‐fat ice cream rheological and microbiological properties and sensory attributes.

## 2 MATERIALS AND METHODS

### 2.1 Preparation of DFP

The pulp of Siwi date (local market, Asyut, Egypt) was extracted from the kernel by boiling in water for 15 min to make the sugars (sucrose, glucose, and fructose) soluble. Afterward, date fibers and pits were recovered through filtration using a 0.2‐mm sieve. The pits were then removed, and the fibers were concentrated by rinsing in 40˚C water. Filtration was performed until the residue was free of sugar, which took approximately five successive rinsings. The residues were then pressed, dried in the oven at 65˚C for 24 hr, and milled in a blender at 5000 rpm to get Siwi date fiber powder (DFP) with <0.2 mm particle size. The concentrate was stored at −18˚C until further analyses (Yangılar, 2015). Siwi date and final DFP were analyzed for fat (Hooi et al. 2004), protein (AOAC, 2000; method 991.20; 33.2.11), total solids (TS) (AOAC, 2000; method 990.20; 33.2.44), and ash content (AOAC, 2000; method 945.46; 33.2.10). Also, total carbohydrate, total dietary fiber, soluble, and insoluble fibers were determined (AOAC, 2000).

### 2.2 Manufacture of LFIC mix

**FIGURE 1:**
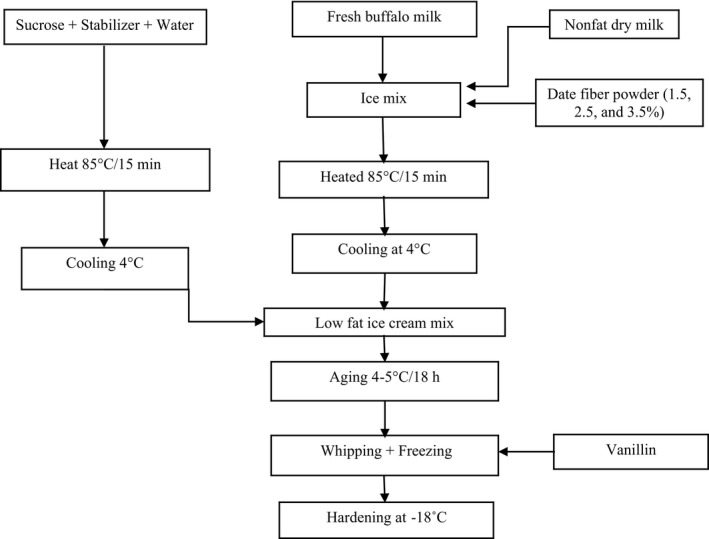
Flow diagram for making low‐fat ice cream (LFIC) using date fiber powder (DFP)

The LFIC mix was formulated to have 3% fat (fat source was buffalo milk, Animal Production Farm, Faculty of Agriculture, Al‐Azhar University), 14% milk solids nonfat (SNF) (skim milk powder, Dairy America, local market), 15% sucrose (local market), 0.3% stabilizer (high viscosity minimum assay 95.5% carboxy methyl cellulose, Misr Food Additives), and 0.1% vanillin (local market). The LFIC mix was produced as shown in Figure 1. A 3kg batch of LFCI was prepared from each formulation. All ingredients used to produce LFIC formulations are shown in Table 1. Three different levels of DFP were used in the LFIC mix as follows: 1.5% (T1), 2.5% (T2), and 3.5% (T3). Fresh buffalo milk, skim milk powder (SMP), and DFP were mixed, heated at 85°C for 15 min, following by cooling at 4°C. Meanwhile, sucrose and stabilizer were solved in 85°C water for 15 min, then cooling at 4°C. After that, both blends were mixed evenly to get the LFIC ready. The LFIC was manufactured three times using DFP.

**TABLE 1:** Composition of Siwi date and date fiber powder (DFP)

Composition (g/100 g)	Siwi date	Date fiber powder
Moisture	12.68	3.89
Ash	1.88	2.85
Protein	1.95	8.93
Fat	4.20	0.80
Carbohydrates	77.89	0.0
Total dietary fibers	13.40	83.30
Soluble dietary fiber	4.30	27.66
Insoluble dietary fiber	9.10	55.53

### 2.3 Analyses of LFIC mix

The ice cream mix was analyzed for relative viscosity (Arbuckle, 1986b) and density (Equation 1). The weight per gallon (lb) was also calculated in the mix as described by Arbuckle (Arbuckle, 1986a) by multiplying the density by the factor of 8.34.

(1) $$\text{Density}\;\left(g/{\text{cm}}^{3}\right)=\frac{\text{Weight}}{\text{Volume}}$$

Some microbiological analyses were also performed on the mix. Total bacterial count (TBC) was enumerated by using the standard plate count technique (Wehr & Frank, 2004). Psychrotrophic bacteria count (PBC) was also enumerated by plating using the SPC procedure and incubating for 10 days at 7°C (Wehr & Frank, 2004). The coliform count was determined on MacConkey broth media, and tubes were incubated at 32°C ± 1°C for 24 hr (Ashenafi, 1990). Yeast and mold count were also enumerated (Wehr & Frank, 2004) using potato dextrose agar media, and plates were incubated at 25°C ± 1°C for 5 days.

### 2.4 Manufacture of LFIC

Mixing all ingredients (Figure 1), aging was done at 4–5°C for 18 hr. The whipping of the mix was applied (Mantematic 3/Cattabriga) at 4–5°C, then freezing. The final LFIC products were stored at −18°C for 30 days and examined for physicochemical, microbiological, rheological, and sensory properties.

### 2.5 Analyses of final LFIC

Density and weight per gallon (lb) were measured in the final LFIC product as described in the mix. The melting resistance of LFIC (Arbuckle, 1986b) and overrun (Arbuckle, 1986b; Gheisari et al., 2020; Muse & Hartel, 2004) was also determined. Additionally, the TBC, PBC, coliform, and yeast and mold were also enumerated in the final LFIC.

The sensory characteristics of LFIC samples were evaluated according to 10–15 trained panelists from the Dairy Science Department, Al‐Azhar University. The LFIC was examined as described by Arbuckle with some modifications (Arbuckle, 1986c). Samples were evaluated for color and appearance (20 points), flavor (30 points), melting quality (20 points), and body and texture (30 points) to have 100 points overall. This experiment was performed in triplicates.

### 2.6 Statistical analysis

Data were analyzed by R software (R x64‐3.3.3). All data were analyzed by ANOVA using a GLM for each variable to study the effect of DFP concentration and storage time on the characteristics of LFIC. Mean separation was done using the least significant difference (LSD) comparison test when significant differences were detected at *p* < .05.

## 3 RESULTS AND DISCUSSION

### 3.1 Composition of Siwi date and DFP

Table 1 illustrates the composition of Siwi date and DFP. The moisture content (on dry weight basis) of the Siwi date was higher (12.68%) than that of DFP (3.89%). The ash and protein contents were 1.88% and 1.95%, respectively, in Siwi date compared to 2.85% and 8.93%, in DFP, while the fat content was 4.2% in Siwi date relative to 0.8% in DFP. The total dietary fiber was 13.4% and 83.3% in Siwi date and DFP, respectively, with a ratio of 2:1 insoluble dietary fiber to soluble dietary fiber. These results are in agreement with those reported in Siwi date syrup and Siwi date powder (Di Cagno et al., 2017).

### 3.2 Formulations of LFIC

Formulations of LFIC were calculated using TechWizard software (Excel‐based formulation software). Table 2 shows the formulations of LFIC using different levels of DFP (1.5%, 2.5%, and 3.5%). The control treatment was made with no DFP for comparison. The formulations were targeted to have 3% fat, 14% milk solids nonfat, 15% sucrose, 0.3% stabilizer, and 0.1% vanillin using different ingredients including fresh buffalo milk, SMP, sucrose, stabilizer, vanillin, water, and DFP. Fresh buffalo milk, SMP, sucrose, stabilizer, and vanillin were almost constant in all treatments with changes in water and DFP contents.

**TABLE 2:** Formulations of low‐fat ice cream (LFIC) mix made with date fiber powder (DFP)

Ingredients	Treatment^1^			
	C	T1	T2	T3
Fresh buffalo milk	46.00	45.60	46.10	46.10
Skim milk powder (SMP)	10.80	10.80	10.75	10.75
Sucrose	15.0	15.0	15.0	15.0
Stabilizer	0.30	0.30	0.30	0.30
Vanilla	0.10	0.10	0.10	0.10
Water	27.80	26.70	25.25	24.25
Date fiber powder (DFP)	0.0	1.50	2.50	3.50
Total	100	100	100	100

^1^ C = control LFIC; TI = 1.5% DFP; T2 = 2.5% DFP; T3 = 3.5% DFP.

### 3.3 Rheological properties of LFIC mix

The data presented in Table 3 exemplified the rheological properties (relative viscosity, density, and weight per gallon) of LFIC mix prepared using DFP at a rate of 1.5%, 2.5%, and 3.5%. Addition of DFP improved the functionality of the mix, which increased the relative viscosity, density as well as weight per gallon compared to control. The relative viscosity increased two times (1.47 in control vs. 3.33 in T1) after adding 1.5% DFP in the LFIC mix. Increasing the DFP concentration to 3.5% led to increasing the relative viscosity, density, and weight per gallon by 5.91, 1.42 g/cm^3^, and 11.88 lb, respectively. Gheisari found that the addition of date to ice cream formulations led to an increase in the viscosity due to the high‐fiber content in date (Gheisari et al., 2020).

**TABLE 3:** Effect of different levels of date fiber powder (DFP) on relative viscosity, density, and weight per gallon of low‐fat ice cream (LFIC) mix

Components	Treatment^1^			
	C	T1	T2	T3
Relative viscosity	1.47 ^d^	3.33 ^c^	4.13 ^b^	5.91 ^a^
Density (g/cm^3^)	1.25 ^d^	1.27^c^	1.35 ^b^	1.42 ^a^
Weight per gallon (lb)	10.42^d^	10.64^c^	11.29 ^b^	11.88 ^a^

^1^ C = control LFIC; TI = 1.5% DFP; T2 = 2.5% DFP; T3 = 3.5% DFP.

^a‐d^ Means in the same row not sharing a common superscript are different (P< .05).

### 3.4 Microbiological analyses of LFIC mix

Data presented in Table 4 illustrate the TBC, PBC, coliform, and yeast and molds count of LFIC mix made with 1.5%, 2.5%, and 3.5% DFP. The TBC and yeast and molds counts of LFIC mix were affected (*p* < .05) by the percentage of DFP. There was a slight increase (*p* < .05) in TBC and yeast and molds counts in LFIC mix with increasing of DFP. The TBC in LFIC mix was 4.37, 4.38, 4.39, and 4.4 log cfu/ml in control T1, T2, and T3, respectively, while yeast and mold counts were 1.43 log cfu/ml in control and T1, 1.44 log cfu/ml in T2, 1.45 log cfu/ml in T3. On the other hand, PBC and coliform were not detected in the LFIC mixes, and this is due to the sanitization environment.

**TABLE 4:** Effect of different levels of date fiber powder (DFP) on the microbiological counts (log cfu/ml) of low‐fat ice cream (LFIC) mix

Microbial type	Treatment^1^			
	C	T1	T2	T3
Total bacterial count (TBC)	4.37 ^b^	4.38 ^ab^	4.39 ^a^	4.4 ^a^
Yeast and molds count	1.43 ^b^	1.43 ^b^	1.44^a^	1.45^a^

^1^ C = control LFIC; TI = 1.5% DFP; T2 = 2.5% DFP; T3 = 3.5% DFP.

^a‐d^ Means in the same row not sharing a common superscript are different (P< .05).

### 3.5 Rheological properties of the final LFIC

The data presented in Table 5 exemplified the density, weight per gallon, and overrun in the final LFIC prepared using DFP. There were significant increases (*p* < .05) in density and weight per gallon (lb) with elevating the concentration of DFP, which is similar to the trend in the mix (Table 3). The density of control LFIC was 0.62 g/cm^3^, and this value increased to 0.80, 0.95, and 1.08 g/cm^3^ after addition of 1.5%, 2.5%, and 3.5%, respectively. The weight per gallon also increased from 5.21 lb in control to 6.71 lb in T1, 7.93 lb in T2, and 9.02 lb in T3. However, overrun decreased (*p* < .05) to 36.87, 29.70, and 24.04 after addition of 1.5%, 2.5%, and 3.5% DFP, respectively, compared to 50.0 in control. The overrun is referred to as the ability of ingredients and mix to retain air bubbles, which revealed that DFP has a lower ability to retain air bubbles.

**TABLE 5:** Effect of different levels of date fiber powder (DFP) on density, weight per gallon, and overrun of low‐fat ice cream (LFIC)

Components	Treatment^1^			
	C	T1	T2	T3
Density (g/cm^3^)	0.62^d^	0.80^c^	0.95 ^b^	1.08^a^
Weight per gallon (lb)	5.21 ^d^	6.71 ^c^	7.93 ^b^	9.02^a^
Overrun (%)	50.0^a^	36.87 ^b^	29.70 ^c^	24.04^d^

^1^ C = control LFIC; TI = 1.5% DFP; T2 = 2.5% DFP; T3 = 3.5% DFP.

^a‐d^ Means in the same row not sharing a common superscript are different (P< .05).

The density, weight per gallon, and overrun of LFIC improved with the addition of DFP. Similar results for control were reported in other studies (Arbuckle, 1986b; Muse & Hartel, 2004). The functionality of fiber could increase water‐binding amplitude and thickness of ice cream, which in turn, led to an increase in the density and weight per gallon of LFIC (Staffolo et al., 2004). It also may be related to the difference in moisture content of LFIC mix. Remarkably, the addition of 3.5% of DFP increases the density and, weight per gallon, and decreases overrun by twofold compared to the control. The interaction between fiber and protein can lead to improvement.

**FIGURE 2:**
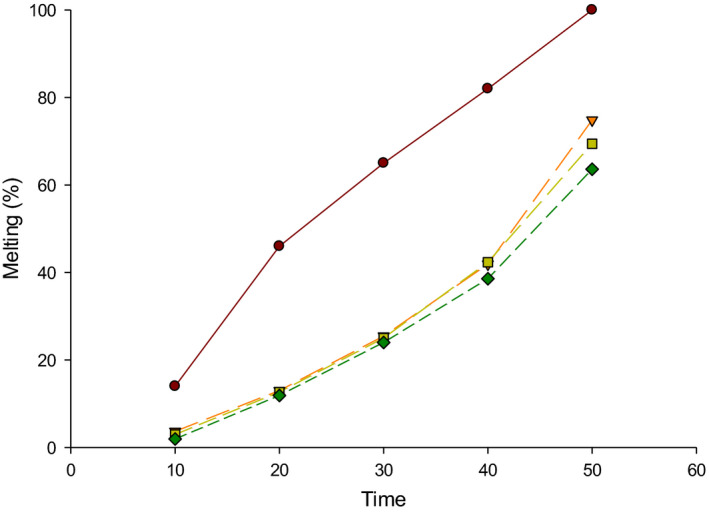
Effect of different levels of date fiber powder (DFP) on melting resistance (%) of low‐fat ice cream (LFIC). C = control (●); TI = 1.5% DFP (▼); T2 = 2.5% DFP (■); T3 = 3.5% DFP (♦)

The melting resistance of LFIC made with DFP is shown in Figure 2. The melting characteristics of LFIC showed significant differences (*p* < .05). The melting property of LFIC after 10 min at 37°C was 3.66%, 2.95%, and 1.92% for the LFIC containing 1.5%, 2.5%, and 3.5% DFP, respectively, while 14% of control ice cream was melted after 10 min. It was expected that increasing the time would lead to elevate the melting area of LFIC. The control ice cream was melting fast as compared to LFIC made with DFP after 10, 20, 30, 40, and 50 min, which means that DFP is increasing the melting resistance. These melting values increased to 41.88%, 42.33%, and 38.95% after 40 min for the LFIC containing 1.5%, 2.5%, and 3.5% DFP, respectively, compared to 82% in control. The control ice cream was completely melted after 50 min, while 74.85%, 69.44%, and 63.30% of T1, T2, and T3, respectively, were melted. The differences in melting resistance between control and DFP ice cream are contributed to freezing points and viscosity of LFIC that was affected by the addition of DFP.

The melting resistance was improved with the addition of DFP. The result shows an inverse relationship between melting resistance and overrun. Similar results were reported in another study (Sakurai et al., 1996). One of fiber functionality is increasing the water‐binding that delays melting point (Staffolo et al., 2004). The amount of air incorporated and fat percentage can affect the melting resistance (Muse & Hartel, 2004).

### 3.6 Microbiological analysis

Table 6 illustrates the means squared and *p*‐values of LFIC made with DFP, while Table 7 shows averages of TBC, PBC, coliform, and yeast and molds counts of LFIC during 30 days of storage. The TBC and yeast and molds counts of LFIC were affected by the addition of DFP during 30 days of storage period at −18°C. There was a slight decrease (*p* < .05) in TBC and yeast and molds counts in LFIC with increasing the percentage of DFP (Table 7). The TBC in all treatments decreased from approximately 4.4 to 4.3 log cfu/ml after 30 days of storage, with control having the lowest TBC. Control samples had also lower counts of yeast and mold than that of LFIC made with DFP. On the other hand, the data observed that PBC and coliform were not detected during the 30 days of LFIC at −18°C.

**TABLE 6:** Mean squares and *p*‐values (in parentheses) of low‐fat ice cream (LFIC) made with date fiber powder (DFP)

Factor	*df*	Total bacterial	Yeast and molds	Flavor	Body & texture	Melting quality	Appearance	Overall
Treatment^1^	3	0.0046 (.01)^*^	0.005 (4e‐7) ^***^	6.8 (.001)^**^	0.61 (.10)	9.0 (5e ‐ 5) ^***^	2.68 (.001)^**^	12.1 (.01)^*^
Time^2^	2	0.0027 (.1)	0.0008 (.051)	10.9 (.0003)^***^	2.07 (.002)^**^	3.9 (.01)^*^	0.43 (.3)	34.9 (.0002)^***^
Replication	2	0.0028 (.09)	0.0004 (.19)	0.94 (.377)	2.24 (.0017)^**^	2.8 (.03)^*^	0.48 (.27)	10.7 (.03)^*^
Treatment x Time	6	0.0005 (.81)	0.0001 (.79)	0.48 (.784)	5.86 (2.5e ‐ 8) ^***^	0.5 (.60)	0.97 (.036)^*^	6.5 (.06)
Error	22	0.001	0.0002	0.927	0.26	0.71	0.35	2.7

^1^ Treatment: C = control LFIC; TI = 1.5% DFP; T2 = 2.5% DFP; T3 = 3.5% DFP.

^2^ Time: 0, 15, and 30 days.

^*^ Statistically significant at *p* < .05; ^**^ *p* < .01; ^***^ *p* < .001.

**TABLE 7:** Effect of different levels of date fiber powder (DFP) on microbiological counts (log cfu/ml) of low‐fat ice cream (LFIC)

Microbial type	Storage (day)	Treatment^a^			
		C	T1	T2	T3
Total bacterial count (TBC)	0	4.36	4.37	4.38	4.39
	15	4.33	4.36	4.37	4.39
	30	4.30	4.36	4.36	4.36
Yeast and molds count	0	1.4	1.43	1.44	1.44
	15	138	1.41	1.43	1.43
	30	1.36	1.41	1.43	1.43

^1^ C = control LFIC; TI = 1.5% DFP; T2 = 2.5% DFP; T3 = 3.5% DFP.

The slight decrease of microbial number during storage time could be related to decrease oxygen and low temperatures that led to damage the viable cells (Senaka Ranadheera et al., 2013). A direct correlation between the TBC and the percentage of fiber could be due to acidity production with additive fiber (do Espírito Santo et al., 2012).

### 3.7 Organoleptic properties

Table 8 presents the sensory properties of LFIC. Flavor, body and texture, melting quality, and appearance of LFIC were affected by the addition of DFP and 30 days of storage period at −18°C. Flavor scores of LFIC made with DFP were slightly lower than control during the 30 days of storage at −18°C. There was a slight decrease in the flavor of LFIC of all treatments during the 30 days of storage, and this decrease was more with increasing the DFP concentration. The lowest flavor score (23.25) was observed in T3 (3.5% DFP), while the flavor of T1 (24.25) and T2 (24.0) was relatively similar to the control (24.25) after 30 days of storage. Additionally, the body and texture scores were slightly lower in LFIC made with DFP as compared to control. The body and texture were 27.58, 25.85, 25.65, and 24.15 in control, T1, T2, and T3, respectively, after 30 days of storage.

**TABLE 8:** Effect of date fiber powder (DFP) on organoleptic properties of low‐fat ice cream (LFIC)

Properties	Storage (day)	Treatment^1^			
		C	T1	T2	T3
Flavor (30)	0	26.77	26.0	25.65	24.35
	15	26.85	25.75	25.25	24.05
	30	24.25	24.25	24.0	23.25
Body and texture (30)	0	25.64	25.91	27.65	27.35
	15	25.14	25.85	26.0	27.65
	30	27.58	25.85	25.65	24.15
Melting quality (20)	0	16.47	17.05	19.05	18.95
	15	15.86	15.91	17.65	17.53
	30	17.2	16.25	18.05	17.91
Appearance (20)	0	16.36	17.41	18.15	17.41
	15	17.23	17.25	17.0	18.05
	30	16.3	16.25	17.58	18.0
Overall scores (100)	0	87.49	88.62	92.75	90.31
	15	87.33	87.01	88.15	89.53
	30	87.58	84.85	87.53	85.56

^1^ C = control LFIC; TI = 1.5% DFP; T2 = 2.5% DFP; T3 = 3.5% DFP.

However, melting quality and appearance improved (*p* < .05) with increasing the level of DFP concentrations as compared to control during storage. The addition of 2.5% and 3.5% DFP in the LFIC mix improved the melting quality and appearance of LFIC relative to control. The melting quality scores of LFIC were 17.2, 16.25, 18.05, and 17.91 in control, T1, T2, and T3, respectively, while the appearance was 16.3 in control, 16.25 in T1, 17.58 in T2, and 18.0 in T3 after 30 days of storage at −18°C. The overall scores of LFIC made with DFP were improved, especially in T2 and T3 (88.15 and 89.53) up to 15 days of storage as compared to control (87.33), while those scores became lower than control after 30 days.

It has been reported that the addition of date pulp to ice cream formulations did not affect the flavor (Gheisari et al., 2020). The lower flavor score of LFIC with the addition of DFP could be related to the growth of ice crystals (Schaller‐Povolny & Smith, 1999). The addition of fiber has decreased the flavor score of ice cream comparing to control (Crizel et al., 2014). It may contribute to acidity values produced from additive fiber during storage time (de Moraes Crizel et al., 2013). The body and texture of LFIC were improved with the addition of DFP that related to decreasing the ice by improving the capacity of water‐binding (Soukoulis et al., 2009). The LFIC made with DFP was acceptable with high overall scores, which was similar to a previous study (Gheisari et al., 2020).

## 4 CONCLUSION

The addition of DFP at a level of 1.5%, 2.5%, and 3.5% improved the density, weight per gallon, and melting resistance of LFIC. Additionally, DFP improved the sensory characteristics of LFIC, including body and texture, melting quality, and appearance as compared to control during 30 days of storage at −18˚C. DFP is a suitable and cheap ingredient that can be used as a valuable source of fiber in ice cream formulations. DFP has more benefits from nutritional and technological aspects. DFP can replace the fat in ice cream formulations to produce LFIC with characteristics relatively similar to those in full‐fat ice cream.

## Data Availability

Research data are not shared.
